# Field studies reveal a close relative of *C. elegans* thrives in the fresh figs of *Ficus septica* and disperses on its *Ceratosolen* pollinating wasps

**DOI:** 10.1186/s12898-018-0182-z

**Published:** 2018-08-21

**Authors:** Gavin C. Woodruff, Patrick C. Phillips

**Affiliations:** 1 Forest Pathology Laboratory, Forestry and Forest Products Research Institute, Tsukuba, Japan; 2 Department of Biology, Institute of Ecology and Evolution, University of Oregon, Eugene, OR USA

**Keywords:** *Ficus*, Fig wasp, *Caenorhabditis*, Natural history, Coevolution

## Abstract

**Background:**

Biotic interactions are ubiquitous and require information from ecology, evolutionary biology, and functional genetics in order to be understood. However, study systems that are amenable to investigations across such disparate fields are rare. Figs and fig wasps are a classic system for ecology and evolutionary biology with poor functional genetics; *Caenorhabditis elegans* is a classic system for functional genetics with poor ecology. In order to help bridge these disciplines, here we describe the natural history of a close relative of *C. elegans*, *Caenorhabditis inopinata*, that is associated with the fig *Ficus septica* and its pollinating *Ceratosolen* wasps.

**Results:**

To understand the natural context of fig-associated *Caenorhabditis*, fresh *F. septica* figs from four Okinawan islands were sampled, dissected, and observed under microscopy. *C. inopinata* was found in all islands where *F. septica* figs were found. *C.i nopinata* was routinely found in the fig interior and almost never observed on the outside surface. *C. inopinata* was only found in pollinated figs, and *C. inopinata* was more likely to be observed in figs with more foundress pollinating wasps. Actively reproducing *C. inopinata* dominated early phase figs, whereas late phase figs with emerging wasp progeny harbored *C. inopinata* dauer larvae. Additionally, *C. inopinata* was observed dismounting from *Ceratosolen* pollinating wasps that were placed on agar plates. *C. inopinata* was not found on non-pollinating, parasitic *Philotrypesis* wasps. Finally, *C. inopinata* was only observed in *F. septica* figs among five Okinawan *Ficus* species sampled.

**Conclusion:**

These are the first detailed field observations of *C. inopinata*, and they suggest a natural history where this species proliferates in early phase *F. septica* figs and disperses from late phase figs on *Ceratosolen* pollinating fig wasps. While consistent with other examples of nematode diversification in the fig microcosm, the fig and wasp host specificity of *C. inopinata* is highly divergent from the life histories of its close relatives and frames hypotheses for future investigations. This natural co-occurrence of the fig/fig wasp and *C. inopinata* study systems sets the stage for an integrated research program that can help to explain the evolution of interspecific interactions.

**Electronic supplementary material:**

The online version of this article (10.1186/s12898-018-0182-z) contains supplementary material, which is available to authorized users.

## Background

Interactions at a broad range of scales structure the organization of biological systems. Within ecology, the biotic environment is a major determinant of the distribution and abundance of both species and communities, and so understanding the origins and maintenance of interspecific interactions is a key goal within the field. Yet, interspecific relationships taken as an aggregate are composed of millions of interactions between individual organisms [1, 2], and the nature of those individuals is in turn strongly dependent upon the interactions of thousands of genetic elements comprising their overall genetic composition [3]. Thus understanding how and why species interact with one another likely depends upon information about the genetic bases of such interactions, of which we currently know very little. A more complete analysis of all of these interactions, from gene to ecosystem, requires the development of study systems in which the power of modern genetic approaches can be used within the context of a compelling ecological circumstance. Here we seek to establish such a system using a newly discovered nematode species that lives in association with the classic fig–fig wasp ecological system [4].

Eukaryotic laboratory model systems have been rightly heralded for their contributions to our understanding of genetics [5–7]. However, only a fraction of their genes are annotated, and thousands of genes remain that have as of yet no known function [8]. Understanding the natural ecological functional context of these genes holds the potential to unlock this mysterious fraction of the genome [8]. Conversely, an understanding of the molecular biology of gene function can be used to inform ecology and evolutionary biology—those interested in the molecular basis of adaptive traits (such as the wing patterns of *Heliconius* butterflies [9], coat color in crows [10], visual sensitivity in fish [11, 12], the timing of maturation in platyfish [13, 14], and beak size in Darwin’s finches [15]), physiological systems that structure species distributions (such as hemoglobin variation underlying altitudinal clines of deer mice [16] and Flowering Locus C and FRIGIDA variation underlying latitudinal clines of *Arabidopsis* [17, 18]), and the underpinnings of host-microbe interactions [1] all need functional genetic tools to address their questions [19]. Indeed, to the extent that genetic elements underlie the distribution and abundance of organisms in space (which in part defines major questions in molecular ecology), such tools will be necessary to empirically test their sufficiency [19]. Are such tools also needed to understand the interspecies interactions that underlie most ecological theory?

Successfully traversing these broad fields requires the development of appropriate study systems—particularly systems wherein questions spanning multiple levels of biological organization can be simultaneously addressed. And although there are systems with compelling ecology and evolution (such as *Heliconius* [20], ants/acacias [21], and Darwin’s finches [22]) and systems with well-established and powerful functional genetics (such as fruit flies [6], yeast [5], and worms [7]), systems with a good knowledge of both are rare. The development of good functional genetics in established ecological systems [8] and/or the development of good ecology in established genetic systems [19] is necessary to bridge these gaps.

A classic system for coevolutionary studies is the fig microcosm [4]. The subject of decades of research efforts [23, 24], this system has revealed important advances regarding mate competition [25–27], sex ratio allocation [26, 28], and the maintenance of interspecific interactions [29], among others. Furthermore, this system entails a textbook mutualism in figs and their associated wasps: figs need wasps for pollination, and wasps lay their eggs in fig ovules [4]. A typical life cycle of a pollinating fig wasp can be defined as follows: (1) one or more winged, female wasps (known as foundresses upon fig entry) enters the fig inflorescence through a specific opening called the ostiole; (2) the foundress either actively or passively pollinates the fig florets; (3) the foundress lays eggs in the ovules (subsequent to egg-laying the foundress typically dies in the fig); (4) wasp progeny develop by feeding on fig ovule tissue; (5) pigment-less, flight-less male wasps emerge first and mate with females that have yet to emerge, and then subsequently cut a hole in the fig to enable female wasp dispersal; (6) female wasps collect pollen, exit the fig, and disperse to another fig to repeat the cycle [4, 24, 30]. Parasitic wasps are also associated with figs and their pollinating wasps [24, 31]. These animals do not pollinate but still lay eggs in the fig, often avoiding the interior fig lumen entirely by laying eggs with a long ovipositor from the exterior fig surface. These parasitic wasps can be fig gallers (which lay eggs into fig tissue), kleptoparasites (laying eggs into existing galls made by pollinating wasps), or parasitoids (laying eggs into developing pollinating wasp larvae) [31]. Furthermore, this system is amenable to experimental manipulation in the field, and evolutionarily-relevant measurements such as the number of seeds, wasp progeny, and wasp foundresses are easily ascertained [4]. Thus, this is a powerful system for investigating a number of fundamental questions in ecological and evolution.

Various nematode species have also been associated with figs and their pollinating wasps. These have long included the plant-parasitic nematode *Schistonchus* (which has recently been re-systematized into the genera *Schistonchus*, *Ficophagus*, and *Martininema* due to paraphyly [32]) and the wasp parasitic nematode *Parasitodiplogaster* [33]. In the past decade, a number of additional nematode species have been discovered to be associated with figs. *Teratodiplogaster* nematodes are close relatives of *Parasitodiplogaster* that are nonetheless morphologically divergent and are presumptive yeast-feeders instead of wasp parasites [34–36]. *Bursaphelenchus sycophilus*, a close relative of fungal feeders, is a likely plant parasite of *Ficus variegata* [37]. The plant-parasitic *Ficotylus* has also been associated with both fig interiors [38] as well as exterior bracts [39]. Additionally, multiple new, morphologically-divergent and highly phenotypically-plastic *Pristionchus* species were discovered in the figs of three different *Ficus* species [40]. These include likely bacteria feeders and nematode predators. This report also included sequencing data suggesting the diplogastrid *Acrostichus* is also associated with figs [40]. Furthermore, multiple plant parasitic nematodes have been observed to infect non-fig areas of the *Ficus* plant (i.e. branches, leaves, roots, etc.; *Meloidogyne*, *Xiphinema*, *Heterodera*, and *Aphelenchoides*, among others [41, 42]). Thus multiple nematode lineages have evolved to thrive in figs through adaptation to various nutrient resource types. In addition, fig-associated nematodes are generally thought to disperse on pollinating female wasps upon their emergence (i.e., step (6) in the generic fig wasp life cycle above [43]). Consistent with this, *Schistonchus* [43, 44], *Parasitodiplogaster* [43], *Teratodiplogaster* [40], *Pristionchus* [40], and *Acrostichus* [40] have all been observed on emerging female pollinating wasps in the field. *Schistonchus caprifici* has been observed with both pollinating and parasitic wasps, although nematodes were more frequently found with pollinating wasps [44]. Furthermore, laboratory chemotaxis experiments showed that *Schistonchus racemosa* is more attracted to female pollinating wasps and their cuticle-derived molecules than those of parasitic wasps and male pollinating wasps [45]. This is all consistent with wasp-mediated dispersal being an essential component of the fig nematode life cycle.

As figs and fig wasps have long been used for evolutionary studies, a classic model system for functional genetics is the nematode *Caenorhabditis elegans*. Like most genetic models, it is easy to rear in the laboratory and is amenable to sophisticated genetic manipulations. Furthermore, the background knowledge concerning its molecular, cellular, and developmental biology is simply vast—we arguably know more about this species than any other metazoan [7]. Recently, the nematode *Caenorhabditis inopinata* (formerly known as *Caenorhabditis* sp. 34), a novel sister species to *C. elegans*, was discovered in Okinawa, Japan [46, 47]. In contrast to *C. elegans*, *C. inopinata* is not a self-fertile hermaphrodite but rather an obligate male/female species [46, 47], like most members of the genus [48]. Furthermore, unlike its close relatives, which thrive in rotting plant material [49], *C. inopinata* was found inside the fresh figs of *Ficus septica* [47]. In addition to its novel ecological context, *C. inopinata* is morphologically divergent from *C. elegans* in multiple respects, despite its phylogenetic position. *C. inopinata* is very long in size, growing to be on average nearly twice as long as *C. elegans*, and this size difference can be largely attributed to postembryonic changes in cell size [47]. In addition, *C. inopinata* develops about twice as slowly as *C*. *elegans*, has much shorter tail spikes than *C*. *elegans*, and harbors enormous sperm that are three times larger in diameter than those of *C. elegans*, among other morphological differences [47]. Furthermore, unlike most fig-associated nematodes, *C. inopinata* is culturable in the laboratory on bacteria-seeded agar plates [46, 47]. As multiple reverse genetic techniques are applicable across the genus [50, 51] as well as in this species [46], *C. inopinata* is particularly well-positioned to connect functional genetics with natural ecology. To this end, here for the first time we describe the natural context of *C. inopinata* through the observation of dissected fresh figs. We examine the extent of *C. inopinata* host specificity with both fig and wasp species, the co-occurrence of worm and fig developmental stages, and the ability of worms to disperse on wasps, with a focus on the implications of these observations for continued studies in both the *C. inopinata* and fig/fig-wasp systems.

## Methods

### Collection sites

**Fig. 1:**
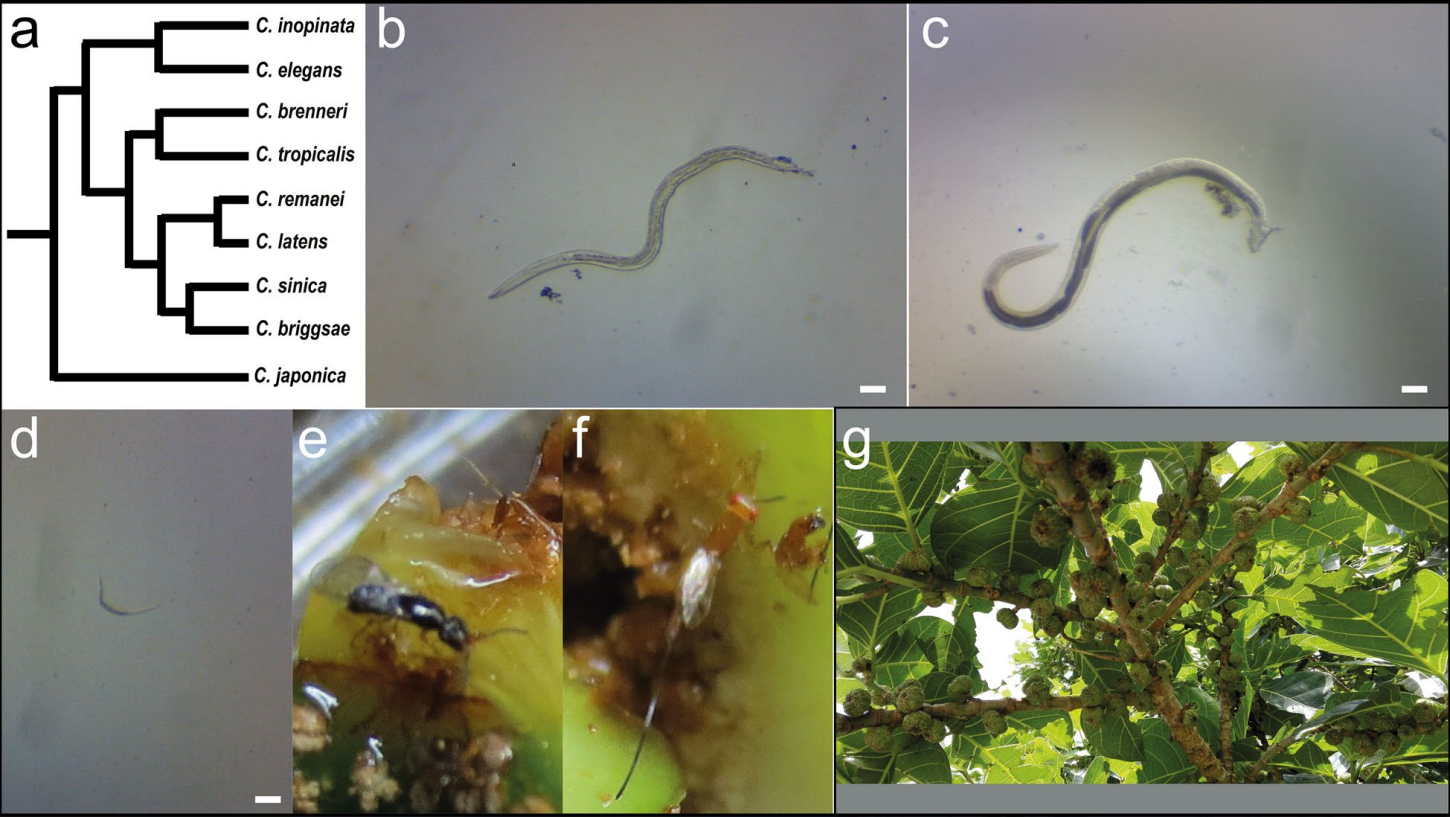
*Caenorhabditis inopinata* is associated with fresh *Ficus septica* figs and fig wasps. **a** A cladogram revealing the evolutionarily relationships of *Caenorhabditis*, following [46, 47]. The fig-associated *C. inopinata* is among the closest known relatives of the important model organism, *C. elegans*. This reduced figure excludes many known species in this group [53]. **b** An adult *C. inopinata* female isolated from a fresh *F. septica* fig. **c** An adult *C. inopinata* male isolated from a fresh *F. septica* fig. **d** A *C. inopinata* dauer larva isolated from a fresh *F. septica* fig. All scale bars in (**b**–**d**) are 100 microns. **e** A female *Ceratosolen* pollinating wasp. **f** A *Philotrypesis* parasitic wasp. **g** A *F. septica* plant

**Fig. 2:**
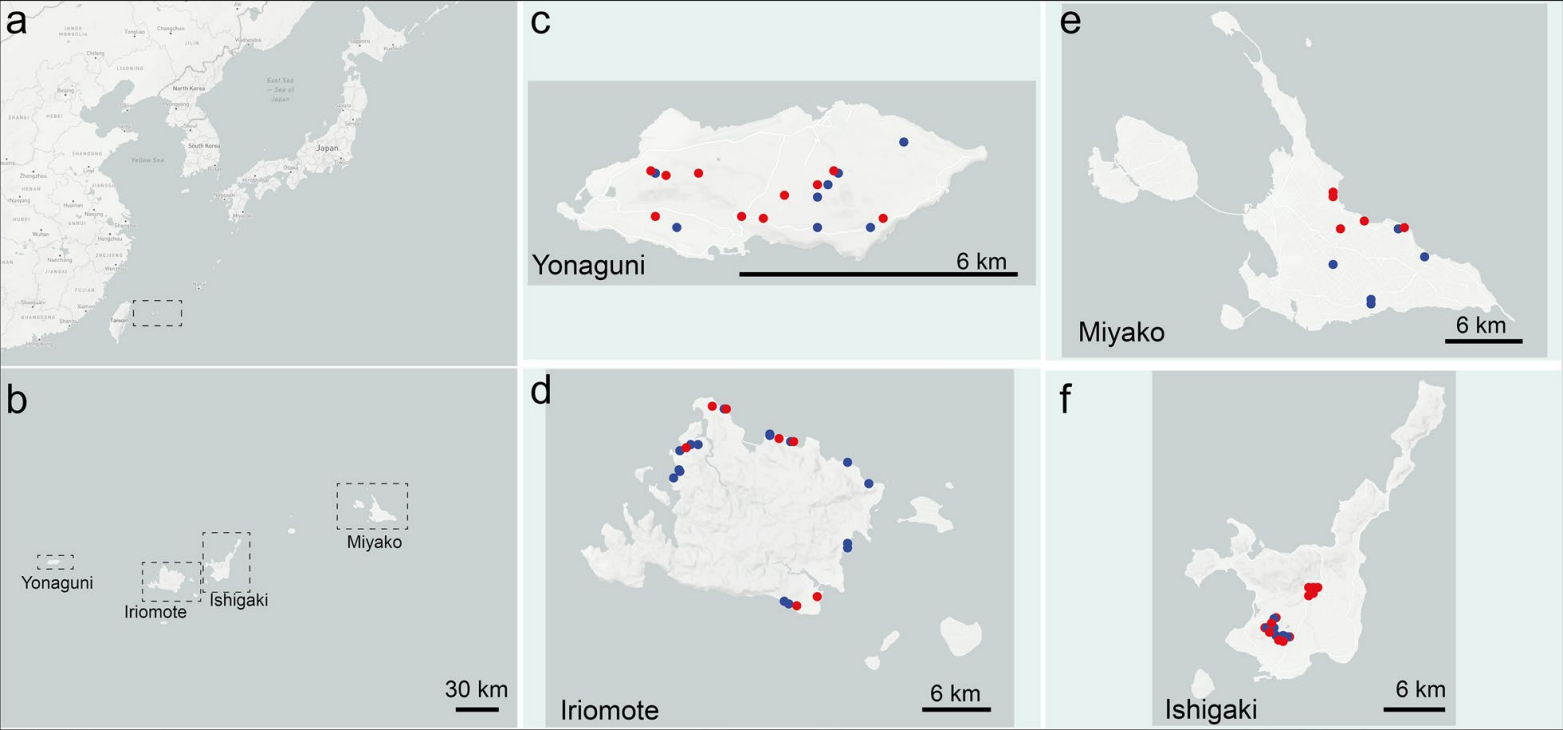
*Ficus septica* fig collection sites in 2016. **a**, **b** Figs were collected in four of the Sakishima Islands (**a**, boxed region) of Okinawa Prefecture, Japan: Yonaguni (**c**), Iriomote (**d**), Miyako (**e**), and Ishigaki (**f**). Blue circles represent positions of *F. septica* plants where *Caenorhabditis inopinata* nematodes were found, and red circles denote positions of *F. septica* plants where *C. inopinata* nematodes were not found in dissected figs

*C. inopinata* was originally isolated from the fresh (that is, not rotting and still attached to the tree) figs of *Ficus septica* on the island of Ishigaki in Okinawa Prefecture, Japan by Natsumi Kanzaki (Fig. 1) [46]. To further probe the natural context of this species, *F. septica* figs were sampled from additional Okinawan islands of Iriomote, Miyako, and Yonaguni (Fig. 2, Tables 1, 2, 3, and Additional File 1). *F. septica* was typically found at the edge of vegetation on roadsides, but sampling was also performed in the public areas of Banna Park (Ishigaki) and Uenootakejoshi Park (Miyako). In May 2015 and May 2016, additional *Ficus* species were also sampled when accessible figs were found. Images revealing geographic position information of sampled plants were generated with Mapbox [52].

**Table 1:** *Caenorhabditis inopinata* occupancy in *Ficus septica* figs in 2016: all sampled plants

Island	All plants^a^	Plants with *C. inopinata*^b^	Plants without *C. inopinata*^c^	All Figs^d^	Figs with *C. inopinata*^e^	Figs without *C. inopinata*^f^
Iriomote	27	19 (0.70)	8 (0.30)	86	49 (0.57)	37 (0.43)
Ishigaki	24	6 (0.25)	18 (0.75)	36	7 (0.19)	29 (0.81)
Miyako	10	6 (0.60)	4 (0.40)	79	17 (0.22)	62 (0.78)
Yonaguni	23	10 (0.43)	13 (0.57)	49	22 (0.45)	27 (0.55)
Total	84	41 (0.49)	43 (0.51)	250	95 (0.38)	155 (0.62)

^a^The number of *F. septica* plants from which figs were picked and dissected. This includes all such plants, regardless of nematode occupancy or pollination status

^b^The number of *F. septica* plants from which at least one dissected fig harbored *C. inopinata*. Fraction of all plants (denominator in Column 2) in parentheses

^c^The number of *F. septica* plants from which no dissected figs harbored *C. inopinata*. Fraction of all plants (denominator in Column 2) in parentheses

^d^The number of dissected *F. septica* figs, regardless of nematode occupancy or pollination status

^e^The number of dissected *F. septica* figs harboring *C. inopinata*, regardless of pollination status. Fraction of all figs (denominator in Column 5) in parentheses

^f^The number of dissected *F. septica* figs that did not contain *C. inopinata*, regardless of pollination status. Fraction of all figs (denominator in Column 5) in parentheses

**Table 2:** *Caenorhabditis inopinata* occupancy and pollination status among *Ficus septica* figs in 2016: plants without *C. inopinata*

Island	Plants^a^	Figs^b^	Pollinated figs^c^	Unpollinated figs^d^	Figs with *C. inopinata*^e^	Figs without *C. inopinata*^f^
Iriomote	8	8	7 (0.87)	1 (0.13)	0	8
Ishigaki	18	20	19 (0.95)	1 (0.05)	0	20
Miyako	4	22	4 (0.18)	18 (0.82)	0	22
Yonaguni	13	16	16 (1)	0 (0)	0	16
Total	43	66	46 (0.70)	20 (0.30)	0	66

^a^The number of *F. septica* plants from which figs were picked and dissected yet none harbored *C. inopinata*. This includes all such plants, regardless of fig pollination status

^b^The number of dissected *F. septica* figs from plants that did not harbor *C. inopinata*, regardless of pollination status

^c^The number of dissected, pollinated *F. septica* figs from plants that did not harbor *Caenorhabditis inopinata*. Fraction of all figs from plants that did not harbor *C. inopinata* (denominator in Column 3) in parentheses

^d^The number of dissected, unpollinated *F. septica* figs from plants that did not harbor *Caenorhabditis inopinata*. Fraction of all figs from plants that did not harbor *C. inopinata* (denominator in Column 3) in parentheses

^e^The number of dissected *F. septica* figs harboring *C. inopinata* from plants that did not harbor *C. inopinata*

^f^The number of dissected *F. septica* figs not harboring *C. inopinata* from plants that did not harbor *Caenorhabditis inopinata*

**Table 3:** *Caenorhabditis inopinata* occupancy and pollination status among *Ficus septica* figs in 2016: plants with *C. inopinata*

Island	Plants^a^	Figs^b^	Pollinated figs^c^	Unpollinated figs^d^	Figs with *C. inopinata*^e^	Figs without *C. inopinata*^f^	Pollinated figs with *C. inopinata*^g^	Unpollinated figs with *C. inopinata*^h^
Iriomote	19	78	78 (1)	0 (0)	49 (0.63)	29 (0.37)	49 (0.63)	0
Ishigaki	6	16	16 (1)	0 (0)	7 (0.44)	9 (0.56)	7 (0.44)	0
Miyako	6	57	55 (0.96)	2 (0.04)	17 (0.30)	40 (0.70)	17 (0.31)	0
Yonaguni	10	33	33 (1)	0 (0)	22 (0.67)	11 (0.33)	22 (0.67)	0
Total	41	184	182 (0.99)	2 (0.01)	95 (0.52)	89 (0.48)	95 (0.52)	0

^a^The number of *F. septica* plants from which at least one fig was picked, dissected, and did harbor *C. inopinata*. This includes all such plants, regardless of fig pollination status

^b^The number of dissected *F. septica* figs from plants that did harbor *C. inopinata*, regardless of pollination status

^c^The number of dissected, pollinated *F. septica* figs from plants that did harbor *C. inopinata*. Fraction of all figs from plants that harbored *C. inopinata* (denominator in Column 3) in parentheses

^d^The number of dissected, unpollinated *F. septica* figs from plants that did harbor *C. inopinata*. Fraction of all figs from plants that harbored *C. inopinata* (denominator in Column 3) in parentheses

^e^The number of dissected *F. septica* figs harboring *C. inopinata* from plants that did harbor *C. inopinata*, regardless of pollination status. Fraction of all figs from plants that harbored *C. inopinata* (denominator in Column 3) in parentheses

^f^The number of dissected *F. septica* figs not harboring *C. inopinata* from plants that did harbor *C. inopinata*, regardless of pollination status. Fraction of all figs from plants that harbored *C. inopinata* (denominator in Column 3) in parentheses

^g^The number of dissected, pollinated *F. septica* figs harboring *C. inopinata* from plants that harbored *C. inopinata*. Fraction of pollinated figs from plants that harbored *C. inopinata* (denominator in Column 4) in parentheses

^h^The number of dissected, unpollinated *F. septica* figs not harboring *C. inopinata* from plants that did harbor *C. inopinata*

### DNA sequencing

*Ficus*, wasp, and nematode species from natural collections (see below) were initially identified via morphological characteristics. Subsequently, DNA was isolated from some ethanol-preserved, *F. septica*-derived wasp and nematode specimens and sequenced to verify genus identity. For wasp samples, preserved animals were washed three times in PBS and subsequently crushed with a pestle in a 1.5 mL Eppendorf tube. DNA was then isolated from the suspension with a Qiagen Blood and Tissue DNeasy kit. For worm DNA samples, preserved single individuals were washed three times in PBS and digested with 5% Proteinase K in Tris–EDTA buffer for 1 h at 58 °C. This solution was immediately used for PCR after a 10 min, 95 °C incubation for enzyme deactivation. For wasp and nematode identification, the mitochondrial cytochrome oxidase I (COI) locus was amplified with primers LCO1490 (5′-GGTCAACAAATCATAAAGATATTGG-3′) and HCO2198 (5′-TAAACTTCAGGGTGACCAAAAAATCA-3′) [54]. PCR reactions were performed with the New England BioLabs Phusion High Fidelity PCR kit. For all reactions this thermocycler program was implemented: 98 °C for 10 min. initial denaturation; 98 °C for 10 s. denaturation; 45 °C for 30 s. annealing; 72 °C for 30 s. extension (37 cycles); 72 °C for 10 min. final extension. Sanger sequencing was performed by Genewiz. Sequences were then queried with BLAST to the NCBI GenBank database to identify closely related taxa (see Additional files 2, 3). COI sequences of known fig-associated nematodes *Parasitodiplogaster salicifoliae* (GenBank accession KP015022) and *Schistonchus guangzhouensis* (now known as *Martininema guangzhouensis* [32]; GenBank accession EU419757), and the marine rhabditid *Litoditis marina* (which was a high BLAST hit for an unidentified nematode species found among our preserved specimens, GenBank accession KR815450) were retrieved from GenBank. Sequences of *Pristionchus pacificus*, *Caenorhabditis japonica*, and *C. elegans* were retrieved from WormBase [55]. The *C. inopinata* COI sequence was retrieved from the genome assembly (https://www.ncbi.nlm.nih.gov/nuccore?term=382947%5BBioProject%5D) [46]. Sequences were aligned with MUSCLE [56].

### Fig dissections and developmental stage classification

Figs were kept refrigerated and dissected < 9 days after sampling. Figs were cut into four pieces in tap water in 60 mm petri dishes. In 2015, figs were only scored for *C. inopinata* presence and fig pollination status. Whereas in May 2016, figs were additionally scored for fig developmental stage, wasp foundress number, and surface nematodes. Unless otherwise noted, the data reported in this study are derived from the larger 2016 set. A fraction of *F. septica* figs (131/250 dissected figs) were initially washed with tap water before dissection in order to interrogate the presence of fig surface nematodes. Dissected figs were then assayed for fig developmental stage, foundress number (in only 169/250 of dissected *F*. *septica* figs), and the presence of *C. inopinata* under a dissection microscope. *Caenorhabditis* nematodes exhibit a stereotypical pharyngeal morphology that was used for species identification [57]. Figs were binned into five stages based on fig wasp development (inspired by the system developed in [23]; Fig. 4a–e): not pollinated (Phase A), pollinated with no apparent developing wasps (Phase B), developing wasp progeny apparent (Phase C), wasp progeny emerging (Phase D), and post-wasp emergence (Phase E). In figs where foundress wasps were unambiguous, they were counted. *C. inopinata* animals were binned into reproductive phase (third larval stage, fourth larval stage, and adult; Fig. 1b, c) or dispersal phase (Fig. 1d). First and second larval stage animals were observed but not noted as they tended to coincide with adult animals and were more difficult to morphologically distinguish with a dissecting microscope (*Caenorhabditis* nematodes are generally thought to have overlapping generations [58]). The dispersing morphotype (Fig. 1d) that dominated later stage figs (Fig. 4f) was confirmed to be *C. inopinata* in the field via pharynx morphology under higher magnification compound light microscopy, DNA sequencing (Additional files 2, 3), and their development into fourth larval and adult *C. inopinata* stages under culture conditions (Fig. 5). As stress conditions can promote both first larval stage arrest and dauer larva formation in *Caenorhabditis* nematodes [59], and the microscopic power necessary to identify key morphological features of dauer larvae [60] was not available in the field, it remains possible that it is early (first or second) larval stages that are dispersing and not the dauer larvae. However, because dauer larvae are the dominant dispersing stage in *Caenorhabditis* and other nematodes [61–63], we assume in this work that this stage is the dauer larva. Regardless, reproductive (non-dauer developmental stages) or dauer *C. inopinata* were noted as “abundant” if ≥ 20 individuals were observed and “rare” if < 20 individuals were observed. Dissected figs were observed under a Nikon SMZ-2 dissection microscope, and pharynx morphologies in young dauer larvae were observed with mounted live specimens under an AmScope M100C-LED compound light microscope.

### Wasp capture, nematode dispersal observations, and fig temperature measurements

Parasitic and pollinating fig wasps emerging from intact *F. septica* figs were caught in a plastic bag (Fig. 1e). These insects were then killed and placed on Nematode Growth Medium (NGM) agar plates seeded with *E. coli* OP50 bacteria [64]. Plates were monitored for disembarking nematodes 3 h and 2 days after plating. Nematodes of a given morphotype were confirmed to be *C. inopinata* via pharyngeal morphology and, in some cases, subsequent development into reproductive phase *C. inopinata* (Fig. 5).

Additionally, interior and exterior *F. septica* figs temperatures were measured with a DeltaTrack needle thermometer. Each interior measurement was performed on one fresh fig on the tree, and 4–5 figs were measured per plant. These data were taken from about 11:30 AM to 1:30 p.m. on May 15, 2016 on Yonaguni Island.

## Results

### *C. inopinata* is found inside the fresh, pollinated figs of *Ficus septica*

*C. inopinata* was originally isolated from a fresh (not rotten) fig of *Ficus septica* in Okinawa, Japan [46]. To further explore the natural context of this species, *F. septica* figs were collected from additional Okinawan islands (Tables 1, 2, 3, Fig. 2, Additional file 1, dissected, and observed under a dissection microscope for the presence of *C. inopinata*. *C. inopinata* nematodes were found on all four islands where *F. septica* was sampled (Tables 1, 2, 3, Fig. 2). In May 2015, *F. septica* was sampled from Ishigaki and Iriomote islands (Additional file 4: Table S4), whereas in May 2016 sampling of figs was expanded to include the islands of Ishigaki, Iriomote, Miyako, and Yonaguni (Tables 1, 2, 3, Fig. 2). Sampling was also attempted on the islands of Okinawa (main island) and Tarama: *F. septica* was not found at all on Tarama, and although *F. septica* was identified on Okinawa main island, figs were not sampled because no easily-accessible figs could be picked. Although the fraction of *F. septica* plants harboring *C. inopinata* in 2016 was largely consistent across islands (G-test of independence p = 0.183, Tables 1, 2, 3, Additional file 4: Tables S1–S3), the fraction of figs with *C. inopinata* showed island-specific differences (G-test of independence p < 0.001, Tables 1, 2, 3, Additional file 4: Table S2). Specifically, the *C. inopinata* fig occupancy was greater in the two western-most islands of Yonaguni and Iriomote than in the eastern islands of Ishigaki and Miyako (Tables 1, 2, 3). These island-specific differences hold even after excluding unpollinated figs (G-test of independence p < 0.001, Additional file 4: Table S3), which were overrepresented on Miyako (Tables 2, 3) and were not expected to harbor nematodes (see below). Additionally, few differences were detected between field work seasons (Tables 1, 2, 3, Additional file 4: Tables S4, S5). However, *C. inopinata* was found less frequently in plants in Ishigaki in 2016 (25% of plants compared to 79% in 2015, Fisher’s exact test p = 0.0022). Also, between-island differences in fig and plant *Caenorhabditis* occupancy could not be detected in 2015 (Fisher’s exact test p = 0.29 and 1, respectively, Additional file 4: Table S4).

*C. inopinata* was originally recovered from a dissected fig. To confirm that *C. inopinata* proliferates in the interior of the fig and not on its surface, *F*. *septica* figs were initially washed in tap water and observed under microscopy before and after dissection. The frequency of *C. inopinata* observed in washed fresh figs is nearly nonexistent (1 out of 131) compared to that of those subsequently dissected (51 out of 131; Fisher’s exact test p < 0.001). Thus, *C. inopinata* is associated with the fig interior and not its surface.

Plants of the genus *Ficus* are renowned for their classic mutualism with pollinating fig wasps [4], and there are a number of *Ficus*-associated nematodes that require such wasps to complete their life cycle [43]. To interrogate whether this might also hold for fig-associated *Caenorhabditis*, *F. septica* figs were also queried for their pollination status, which can be ascertained by the presence of developing seed or pollinating wasp progeny. In both field work seasons, *C. inopinata* animals were never observed in unpollinated *F. septica* figs (Tables 2, 3; in 2015, 0/28 unpollinated figs harbored *C. inopinata*). Thus, *C. inopinata* likely requires pollinating fig wasps in order to thrive.

**Fig. 3:**
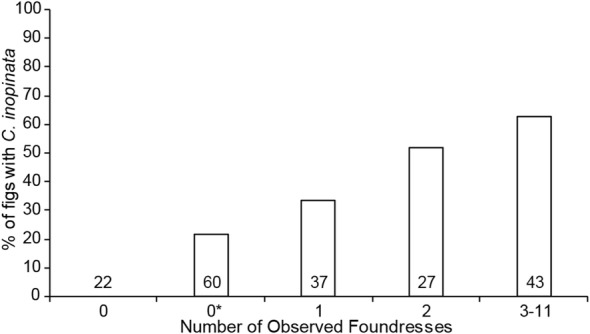
*Caenorhabditis inopinata* is more likely to be found in figs with multiple foundresses. Graphed are the percentages of dissected *F. septica* figs harboring *C. inopinata* by *Ceratosolen* pollinating foundress wasp number. “0*” includes figs that were pollinated but no foundress wasps were seen, whereas “0” notes figs that were not pollinated. Numbers above the x-axis represent fig sample sizes

In addition to pollination status, the number of foundress pollinating wasps per *F. septica* fig was noted. Typically, female pollinating wasps enter the fig, pollinate it, lay eggs in the fig ovules, and die [4]. In a number of cases, a given fig can have multiple foundresses, which can have profound impacts on wasp population dynamics [27, 28, 65]. Indeed, it was observed that the frequency of *C. inopinata* increases with foundress wasp number (Fig. 3, Additional file 4: Table S6). The mean foundress number per fig was more than twice as high in figs with *C. inopinata* (2.8 wasps, SDM = ± 1.3, N = 72) than in those without (1.1 wasps, SDM = ± 0.83, N = 97; Mann–Whitney U p < 0.001; see Additional file 4: Figure S1 for the distribution of observed foundresses across pollinated and unpollinated *F. septica* figs). Thus, higher foundress number is associated with *C. inopinata* fig occupancy, suggestive that these nematodes disperse on pollinating fig wasps.

### *C. inopinata* reproduces in early phase figs and disperses in late phase figs

**Fig. 4:**
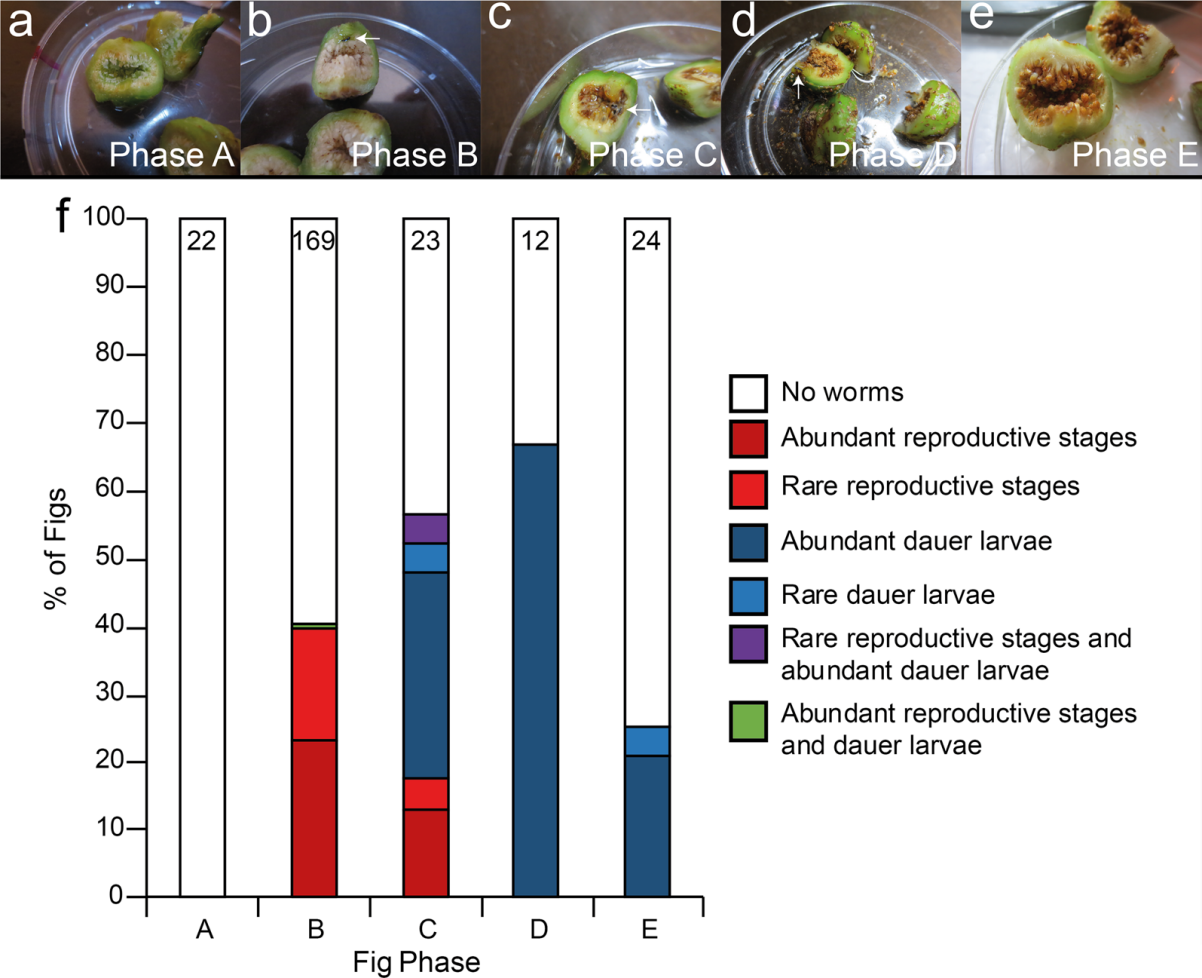
*Caenorhabditis inopinata* proliferates in early phase figs and disperses in late phase figs. **a**–**e** Dissected figs were binned into five developmental stages based on wasp presence and developmental progression: **a** not pollinated (Phase A), **b** pollinated with no apparent developing wasps (Phase B, arrow noting foundress pollinating wasp), **c** developing wasp progeny apparent (Phase C), **d** wasp progeny emerging (Phase D, arrow noting emerging wasp progeny), and **e** post-wasp emergence (Phase E). The presence of abundant (≥ 20 individuals) or rare (< 20 individuals) reproductive stage (not dauer larvae) or *C. inopinata* dauer larvae were noted in each dissected fig (see methods). **f** Frequency of observed *C. inopinata* developmental stage by fig developmental stage. Reproductive *C. inopinata* (i.e., developmental stages that are not dauer larvae) predominates in Phase B and Phase C figs, whereas *C. inopinata* dauer larvae dominate in Phase D and Phase E figs. *C. inopinata* was not observed in figs that were not pollinated. The number of figs dissected per stage is noted at the top of each bar. Reproductive stage and dauer *C. inopinata* frequencies were different between fig stages (G-test of independence p-values < 0.001 for both adult and dispersal types). Nematodes from the fig interior were used for all of these observations. Fisher’s exact test p-values for all pairwise comparisons can be found in Additional file 4: Tables S7, S8

*Caenorhabditis* nematodes can undergo alternative developmental trajectories depending on environmental conditions [66, 67]. If conditions are favorable, animals develop into adults capable of reproduction. But in crowding, starvation, or otherwise stressful conditions, animals develop into the long-lived, stress-resistant dauer larva [66]. It is this dauer stage that is used for dispersal to new food sources in the wild [67]. Previous investigations of fig-associated nematodes have measured the frequency of given nematode developmental stages across fig developmental stages to infer natural histories [40, 43]. To this end, dissected *F. septica* figs were binned into five developmental stages based on wasp presence and development (Fig. 4a–e; inspired by the system developed in [23]): not pollinated (Phase A, Fig. 4a); pollinated with no apparent developing wasps (Phase B, Fig. 4b); developing wasp progeny apparent (Phase C, Fig. 4c); wasp progeny emerging (Phase D, Fig. 4d); and post-wasp emergence (Phase E. Figure 4e). Then, figs were assayed for the presence of rare (< 20 individuals) or abundant (≥ 20 individuals) *C. inopinata* reproductive stage (non-dauer larva developmental stages; Fig. 1b, c) or dauer larval stage (Fig. 1d) animals. Figure 4f summarizes the results, and it is clear that reproducing *C. inopinata* dominate early phase figs. Additionally, *C. inopinata* dauers are not found in early phase figs and rather are only found in late phase figs that are associated with emerging wasp progeny. Furthermore, subsequent DNA sequencing using fixed *Ficus*-derived specimens revealed that these dispersal larvae share near identical sequence similarity to sequence retrieved from the *C. inopinata* genome assembly (Additional files 2, 3), suggestive of identical species status. This distribution of nematode developmental stages then suggests a life cycle wherein nematode founders are dispersed by pollinating wasps, proliferate within the early phase figs, and then generate dispersal forms upon the emergence of wasp progeny.

### *C. inopinata* is observed on *Ceratosolen* pollinating wasps but not *Philotrypesis* parasitic wasps

**Fig. 5:**
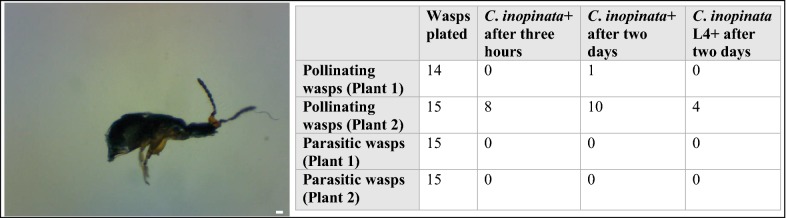
*Caenorhabditis inopinata* is found traveling on pollinating fig wasps but not parasitic wasps. Left, a dispersal *C. inopinata* nematode has dismounted from a pollinating *Ceratosolen* female fig wasp that has been placed on a petri dish. The scale bar represents 10 microns. Right, a table describing wasp carrier data. Fig trees tend to fruit synchronously within a plant but asynchronously between plants [19]. In 2016, two *Ficus septica* plants were observed to harbor figs with actively emerging fig wasps. Emerged fig wasps were caught in a plastic bag, killed, and placed onto agar plates. Plates were subsequently monitored for dismounting *C. inopinata* 3 h and 2 days later. Here, numbers represent the number of plated wasps with disembarking *C.* sp. animals. *C. inopinata* animals were never seen dismounting from parasitic wasps despite their habitat sharing with pollinating wasps harboring *C. inopinata*. “L4,” *C. inopinata* animals at the fourth larval stage of development

**Fig. 6:**
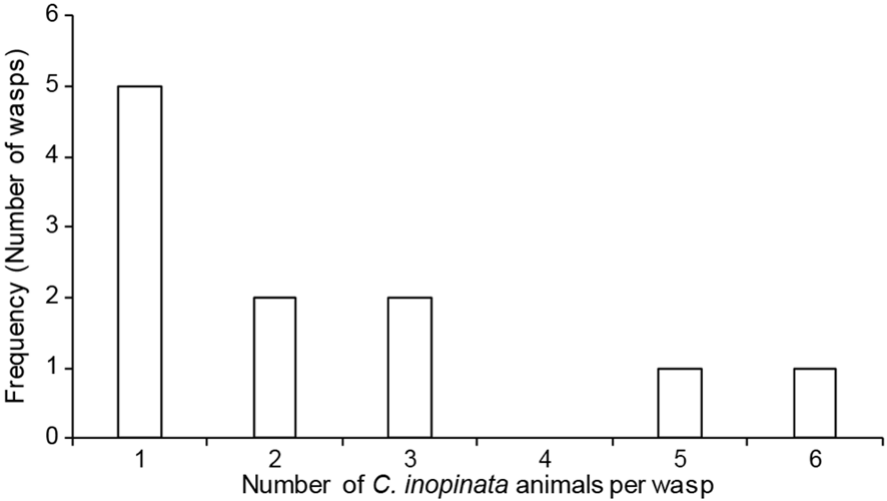
Few *Caenorhabditis inopinata* nematodes dismount from *Ceratosolen* pollinating wasps. In the wasp plating experiment described in Fig. 5, the number of *C. inopinata* animals per wasp were also counted. This histogram reveals that of the pollinating wasps that carried *C. inopinata* (11/29), most carried only one to a few individuals

To confirm the dispersal of *C. inopinata* by fig wasps, emerging *Ceratosolen* pollinating wasps and *Philotrypesis* parasitic wasps were caught in a plastic bag, killed, and placed onto agar plates. Plates were then subsequently monitored at 3 h and 2 days later for the presence of *C. inopinata* nematodes. *C. inopinata* was observed traveling on pollinating wasps (11/29 wasps; Fig. 5) but was never observed on parasitic wasps (0/30 wasps; Fig. 5). Of the 11 wasps harboring *C. inopinata*, there was a median of 2 worms per wasp (range 1–6; Fig. 6). This was despite both species of wasps emerging from the same figs and the same plant. Thus, *C. inopinata* disperses on *Ceratosolen* pollinating fig wasps, and furthermore, *C. inopinata* may host-seek within the fig in order to find a preferred carrier.

### *Caenorhabditis* has only been found in *F. septica* figs among Okinawan *Ficus*

A number of *Caenorhabditis* species are associated with a variety of plant substrates [68, 69]. However, pollinating fig wasps tend to be associated with only one or two species of *Ficus* [4, 70], which suggests that fig wasp-associated *Caenorhabditis* may also be limited to specific *Ficus* species. To determine if this is so, figs from additional Okinawan *Ficus* species were sampled. Of the nine *Ficus* species reported to be in the sampling locales [71], four species were found with fresh figs aside from *F. septica* (Table 4). No figs aside from *F. septica* were found to contain *C. inopinata* nematodes (Table 4), despite some of these species being known to harbor multiple nematode groups [35, 72]. Thus, this particular fig-associated *C. inopinata* is possibly a host specialist and restricted to one species of *Ficus*, however more extensive sampling is required to confirm this association.

**Table 4:** *Caenorhabditis* has not been observed in *Ficus* species other than *Ficus septica*

*Ficus* species	Figs dissected^a^	Figs with *C. inopinata*^b^	Pollinated figs^c^	Plants sampled^d^
2015				
*F. superba*	10	0	–	1
*F. microcarpa*	15	0	15	1
*F. erecta*	15	0	3	1
2016				
*F. variegata*	10	0	7	2
*F. microcarpa*	25	0	25	2
*F. erecta*	36	0	36	2

*Caenorhabditis inopinata* has not yet been observed in *Ficus* species other than *Ficus septica*. Non-*F. septica* figs were dissected in May 2015 and May 2016. There have been eight species of *Ficus* aside from *F. septica* reported on these islands [52]. *Caenorhabditis inopinata* was not observed in five of these (*F. caulocarpa*, *F. ampelas*, *F. benguetensis*, and *F. virgata* figs were not found). “–” = not recorded

^a^The number of dissected figs, regardless of nematode occupancy or pollination status

^b^The number of dissected figs harboring *Caenorhabditis inopinata*, regardless of pollination status

^c^The number of dissected figs that were also pollinated

^d^The number of *Ficus* plants from which figs were picked and dissected. This includes all such plants, regardless of nematode occupancy or pollination status

### *F.* septica figs harbor interior temperatures that are comparable to *C. inopinata* lab-rearing temperatures

**Fig. 7:**
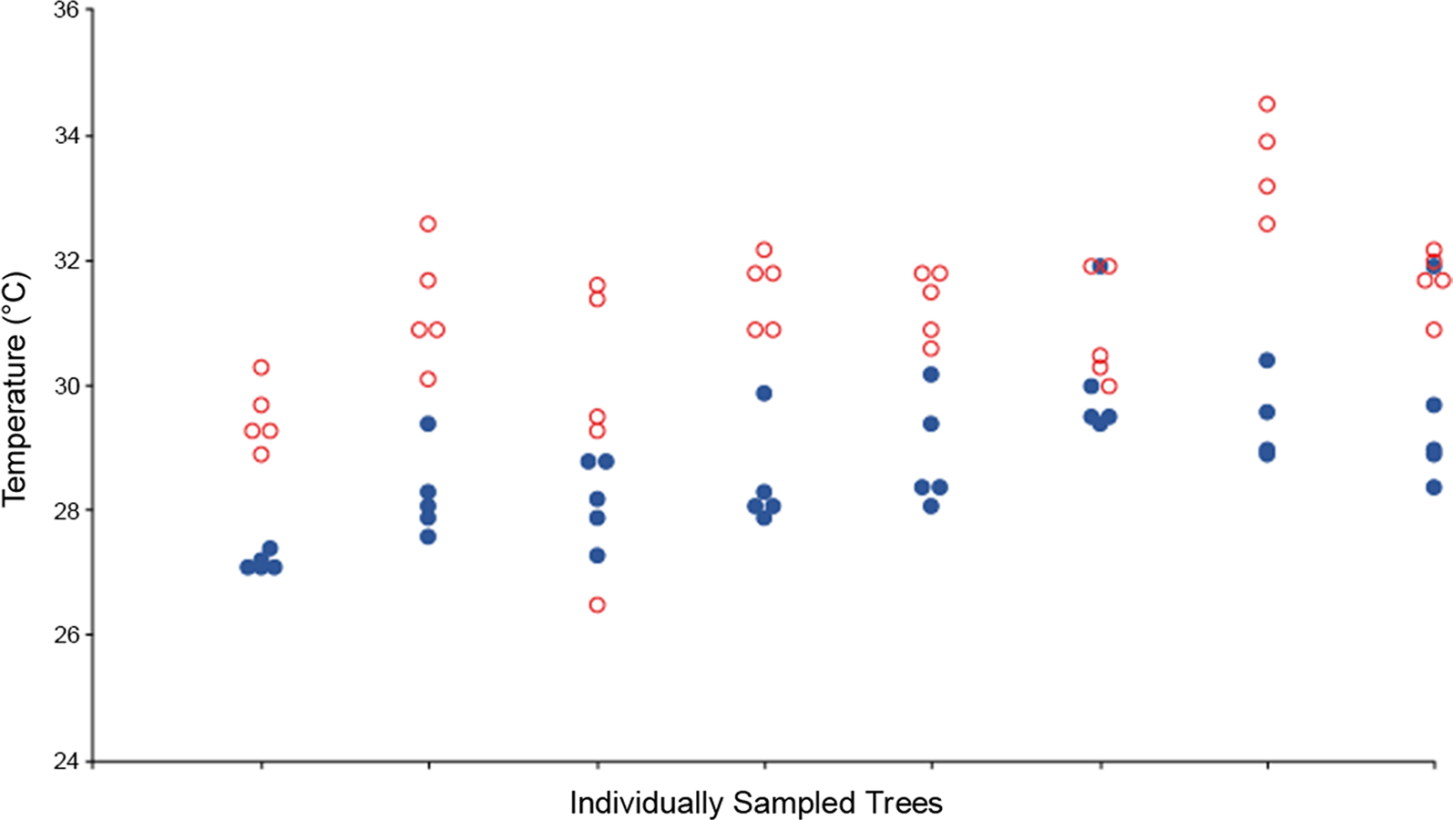
Ambient and interior live *F. septica* fig temperatures. Live *F. septica* figs interiors were measured on eight plants within 1.5 h in the midday. Open red circles represent exterior temperatures, whereas solid blue circles denote interior fig temperatures. Fig interiors were on average 2.4 °C cooler than exterior temperatures (t-test p-value < 0.001)

The environmental parameters defining *Caenorhabditis* ecological niche space are nearly entirely unknown [68]. Among these, temperature influences a multitude of life history traits in *Caenorhabditis*, including survival and reproductive rate [73, 74], as well as the dauer entry switch [75]. To further understand the context of wild *C. inopinata*, interior *F. septica* live figs and exterior ambient temperatures were measured (Fig. 7). Interior fig temperatures (mean = 28.7 °C, SDM = ± 1.2, n = 39) were on average 2.4 °C cooler than exterior temperatures (mean = 31.1 °C, SDM = ± 1.5, n = 39, t-test p-value < 0.001). Interior fig temperatures were comparable to laboratory rearing conditions of *C. inopinata*, wherein the temperature of 25 °C [47] was utilized. Regardless, these observations provide a unique snapshot into the natural context of *C. inopinata*. Future estimates of additional natural environmental parameters will be essential in informing hypotheses regarding the evolution and ecology of these organisms (i.e., what environmental factors are most relevant for fitness, divergence, and speciation in nematodes? [68]).

## Discussion

The intricacy of the fig microcosm has facilitated decades of evolutionary and ecological field studies [4, 30]. It harbors a plethora of diverse interspecific interactions: the fig-pollinating wasp mutualism; fig-ant mutualism [76]; fig-nonpollinating wasp parasitism [31]; nematode-wasp parasitism [43]; fig nematode-fig parasitism [37, 32]; and moth-fig parasitism [77]. Figs are also a key resource for over a thousand bird and mammal species, who in turn aid in seed dispersal [78]. As a consequence of this microcosm complexity, this remains an influential and active system for study in ecology and evolution [79–82]. However, none of the species in these communities are particularly amenable to functional genetics and laboratory studies—both of which are crucial for refining the explanatory power of evolutionary science. Conversely, as thousands of genes in multiple long-standing eukaryotic laboratory model systems have no known functions [8], it is likely that their natural ecological contexts (which have often been neglected) will be needed to thoroughly understand their genomes. As a consequence, there have been calls to integrate ecological, evolutionary, and functional genetic approaches [8, 19]. Here, we have described the natural history of *C. inopinata*, a close relative of the model genetic organism *C. elegans*. What has been observed in this *Caenorhabditis* study, together with the known biology of the fig microcosm, can then be used to inform hypotheses regarding the evolution of interspecific relationships in both systems.

*Caenorhabditis* species typically proliferate on rotting plants and disperse on invertebrate carriers. And although the features defining niche specialization in this group remain uncertain, it seems clear that there is variation in its extent. Some species appear limited in their geographic range (*C. sinica* has only been found in east Asia [83]), whereas others are globally distributed [68]. Interspecific variation in seasonal predominance of wild populations has been observed, consistent with variation in fitness at different temperatures [69]. Furthermore, different *Caenorhabditis* species have been found associated with different bacterial communities [84], consistent with variation in bacterial preference [85]. There is also interspecific variation in the extent of dispersal carrier specificity. Some *Caenorhabditis* species are promiscuous in their choice of carrier; *C. elegans* has been found on snails, slugs, isopods, and myriapods [69]. Other species (such as *C. japonica*, *C. angaria*, and *C. drosophilae*), despite intensive sampling, have only been observed dispersing on one insect species in a highly host-specific manner [68, 86]. The existence of *C. inopinata* in the fresh figs of a single species of *Ficus* and observations of its dispersal via pollinating wasps reveals a dramatic shift in substrate from rotting plants to fresh figs. This intimate coupling further reveals an added instance of carrier host-specificity in this group. Further, this niche shift has coincided with extreme morphological and developmental divergence [46, 47], suggesting that this change in natural history has promoted the evolution of novelties within this species. How does the move to the fig microcosm promote such change and otherwise influence their biology?

Nematodes have evolved to live in association with the fig microcosm at least nine times independently (Additional file 4: Table S9; [37, 39, 40, 87]), and in most cases, the evolution of fig-association co-occurs with dramatic changes in nutrient resource and morphology. *Bursaphelenchus sycophilus* is a fig parasite that evolved from fungal-feeders [37], and *Parasitodiplogaster* nematodes are fig wasp parasites that likely evolved from bacteria-feeders [33, 87]. Upon becoming associated with figs, the *Teratodiplogaster* and *Parasitodiplogaster* clades rapidly diverged in mouth morphology, consistent with their divergent nutrient sources (fungi and wasps, respectively) [34, 87]. In *Pristionchus*, the evolution of fig-association is connected with dramatic plasticity in mouth morphologies, with a single species having up to five different morphotypes [40]. And *Caenorhabditis* reveals rapid changes in morphology upon fig-association, with *C. inopinata* being nearly twice as long as its close relatives [46, 47]. Furthermore, in most of these fig-associated clades there are tight relationships both between nematode development and fig development [43] as well as between nematodes and fig wasps, which are utilized for dispersal or nutrient resources [33, 40, 43, 44]. The fig then represents a microcosm where functional diversification in morphology, ecology, behavior and developmental-decision making has occurred repeatedly throughout nematode phylogeny. Thus, in the case of *C. inopinata*, it may be unsurprising that its biology so divergent from its close relatives.

Because *C. inopinata* has only been observed dispersing on pollinating fig wasps (Fig. 5), it might be expected that they share similarities in population dynamics. Both pollinating wasp and *C. inopinata* founding populations were observed to be quite small [a median of two foundress wasps per fig (Fig. 3) and two dispersing *C. inopinata* per wasp (Fig. 6)], consistent with previous observations of inbreeding in pollinating wasps [26, 88]. Variation in founder population size and its inbreeding effects have been shown to have consequences in local mate competition and sex ratio allocation in fig wasps [26, 27]. This may then also hold for *C. inopinata*, although it is possible that resource availability is different for nematodes (probably bacterial food) and wasps (fig ovules). Male/female *Caenorhabditis* species tend to be incredibly diverse with enormous population sizes, and *C. brenneri* is among the most diverse eukaryotes known [89]. The expected inbreeding in *C. inopinata* should reduce diversity, as has been seen in *C. japonica*, another *Caenorhabditis* male/female species with high host-specificity [90]. The selfer *C. elegans* displays reduced diversity, low global population structure, yet high local structure [91, 92]. This is consistent with a boom-and-bust natural history with high migration and largely clonal local populations initiated by single founders [61, 67, 68]. As *C. inopinata* is dispersed by wasps that can migrate over long distances while exhibiting small founder populations (Fig. 6), they may have more population genetic features in common with selfing lineages than expected of a typical gonochoristic *Caenorhabditis* species.

In contrast to its close relatives, *C. inopinata* populations are likely to be highly influenced by their fig wasp hosts. The extent of spatial population genetic structure of pollinating fig wasp species appears to vary between species. However, it is generally thought that wasps are capable of migrating long distances [93] with some species capable of dispersing over 100 km [94]. Consistent with this, multiple population genetic studies have revealed little spatial structure in pollinating fig wasp species [95–97], with one species (*Valisia javana*) revealing no isolation by distance over 1000 km of southeast Asia [97]. Lack of spatial structure among plant parasitic *Schistonchus caprifici* nematodes among Turkish [98] and southern European [99] populations are consistent with these observations. However, spatial structure is observed in some fig wasp species [100–102], and furthermore, pollination of the same fig species by different wasp species is common, with some figs being pollinated by up to four wasp species [103]. Notably, in the past decade, population genetic studies of *F. septica* and its pollinating *Ceratosolen* wasps themselves have been undertaken across Taiwan and the islands of southeast Asia [95, 100, 102]. Although earlier reports have noted *Ceratosolen bisculatus* as the only pollinator of *F. septica* in Okinawa [71], these studies have found that four species of *Ceratosolen* pollinate *F. septica* across Taiwan, the Philippines, and Okinawa [100, 102]. These pollinators spatially overlap with analogous populations of *F. septica*, revealing a potential example of incipient local co-diversification [102]. These *Ceratosolen* species vary in pigmentation color (with some species black and others yellow), but only one black pollinator species was recovered in Okinawa (“*Ceratosolen* n. sp. 3” [102]). No yellow *Ceratolosen* species were observed in our figs, but additional sequencing of multiple wasps must be carried out in order to determine the extent of wasp species diversity among Okinawan *F. septica* figs. In any case, further molecular characterization of fig, wasp, and nematode diversity [which sequencing suggests includes at least *Martininema* and an undescribed rhabditid or diplogastrid in this system (Additional files 2, 3)] will be needed to fully understand the natural context of *C. inopinata* and the influence of interspecific interactions on its population dynamics.

*C. inopinata* also displays differences in developmental timing and developmental decision-making from their close relatives [47]. Their developmental rate is very slow compared to its close relatives [47], and dauer larvae (an alternative developmental trajectory favored under stress and dispersal conditions) are rarely seen in laboratory populations. Here, we find that reproductive stage, non-dauer animals are enriched in early phase figs and dauer larvae are found in late phase figs (Fig. 4). It was not possible to absolutely confirm that these were morphological dauer larvae due to limitations in microscopy in a field setting. However, given that nearly all *Caenorhabditis* observed on invertebrate carriers are in the dauer stage [67], it is likely that animals found in older figs and fig wasps were indeed dauer larvae. Given that figs typically take weeks to develop [30], and that *C. inopinata* disperses on pollinating wasps to travel to new figs, it is reasonable to suspect that their divergence in developmental timing and decision-making are related to these features of fig biology. Although it is unclear how many generations are produced within a single fig, *C. inopinata* may have faced selective pressure to slow its developmental rate in order to match progeny production with the timing of wasp emergence. Further, given that dispersal on pollinating wasps is likely critical for *C. inopinata* propagation, the decision to enter into dauer may be more dependent on fig and/or wasp chemical cues than those related to stress and population density, which would explain their rarity in laboratory rearing conditions. In addition, fig-associated nematodes often disperse on fig wasps at specific stages in their development: *Schistonchus* fig parasites disperse at the fourth larval stage, and *Parasitodiplogaster* fig wasp parasites disperse at the dauer larval (or infective juvenile) stage [43]. Thus the modulation of developmental timing and decision-making is likely a common adaptation among fig-associated nematodes.

The impact of *C. inopinata* on fig and fig wasp fitness remains an open question. Unlike the fig parasite *Schistonchus* [32] and the wasp parasite *Parasitodiplogaster* [33], *C. inopinata* is unlikely to inflict direct harm on figs or wasps as a parasite. This is because *C. inopinata* maintains its typical *Caenorhabditis* pharyngeal morphology throughout the reproductive stages observed in fresh figs (plant parasitic nematodes typically have pharyngeal stylets [104]), and proliferative animals have not yet been associated with wasps (Figs. 4, 5). As a particle feeder, it is possible *C. inopinata* eats *Ficus* pollen, thereby affecting host fitness. This seems unlikely, however, as *C. elegans* cannot ingest particles greater than 4 microns in diameter [105], and *Ficus* pollen tends to be larger than this on average [106]. *C. inopinata* may affect pollinator wasp fitness through phoresy by somehow adversely affecting pollinating wasp travel across figs. Considering the size of *C. inopinata* dauer larvae (Fig. 1), the pervasiveness of phoresy as a dispersal strategy [107], and the contingency of worm success on wasp success in this case, a large cost to wasp dispersal ability also seems unlikely. Instead, *C. inopinata* more likely impacts host fitness indirectly through bacteriovory. Its impact may then be similar to that of *Pristionchus*, *Acrostichus*, and *Teratodiplogaster*, which are other microbial-feeding nematodes which have been observed in figs [36–38, 40]. Microbes harmful or beneficial for fig and wasp fitness could be a major food resource for *C. inopinata*. Ants similarly impact fig fitness by discouraging non-pollinating wasps from colonizing figs and are associated with decreased fig herbivory [76]. As measures of fig and wasp fitness (number of seeds and foundress progeny, respectively) are easily obtained [4], and contemporary metagenomic tools can define microbial communities [1], the interplay between *C. inopinata* activity, microbial communities, and host fitness should be able to be interrogated in the future. As our understanding of the *Caenorhabditis*-associated microbiota is rapidly increasing [84, 108, 109], this affords an exciting opportunity for future research.

Notably, *C. inopinata* was found dispersing on pollinating *Ceratosolen* wasps, and not *Philotrypesis* parasitoid wasps emerging from figs of the same tree (Fig. 5). In contrast to pollinating wasps, who must enter the fig to lay eggs, *Philotrypesis* wasps do not enter the fig and use long ovipositors to lay eggs from the fig exterior [110]. This suggests that dispersing *C. inopinata* must discriminate within the fig to find the appropriate carrier. This would likely be a novel behavior, as its close relatives are not fig-associated and tend to be promiscuous in carrier choice [69] (although some preferences in *Caenorhabditis remanei* have been noted [111]). The more distantly-related *C. japonica* has been shown to have behavioral preferences for its shield bug host [112], and similar findings have been shown for *Pristionchus* nematodes and their host beetles [113]. In addition, it is important to note that the extent of species diversity among these Okinawan *Ceratosolen* wasps is still unknown, and as a consequence, it remains unclear if *C. inopinata* reveals preferences among *Ceratosolen* species if present. Furthermore, as nematode dispersal can occur in the wasp hemolymph [43], and since wasp dissections have not yet been performed in this system, how *C. inopinata* interacts with the wasp in transit remains an open question; also, our observations do not address the possibility of wasp necromeny in *C. inopinata*, which may occur in the fig-associated *Parasitodiplogaster* [33, 43]. The lack of wasp dissections (in addition to small sample sizes) among our observations here also has possibly led to underestimates of *C. inopinata* load on both pollinating and parasitic wasps (Figs. 5, 6). In any case, as mentioned above, tight associations with fig wasps is widespread among fig-associated nematodes. Nematode occupancy biases on pollinating wasps relative to parasitic wasps have been observed in the fig-associated parasitic *Schistonchus* and *Parasitodiplogaster* nematodes [43, 44], although parasitic wasps can carry nematodes [44, 114]. This typical preference for pollinating wasps has been recapitulated in a laboratory framework with *Schistonchus* using traditional chemotaxis assays with wasp-derived volatiles and cuticular hydrocarbons [45]. Similar studies could be extended to the culturable *C. inopinata* to interrogate the genetic basis of novel behaviors.

## Conclusion

The elegance of contemporary molecular biology resides in the explanatory power generated by conceptual continuity across multiple hierarchical levels [115] (also known as vertical integration [116]). Such continuity is rarely found in evolutionary science—it remains unclear how the disparate pieces of population-level processes, environmental effects, developmental events, and historical contingencies interact to generate diversity in nature. Here, we described the natural history of a close relative of *C. elegans* that is associated with figs and fig wasps. The fig–fig wasp system is a legendary study system in evolution and ecology, and *C. elegans* is a legendary one in model systems genetics. Here then is a serendipitous convergence of research organisms that can facilitate the conceptual connection of their respective disciplines. The functional genetics of *C. inopinata* has the potential to inform the molecular basis of how ecologically-relevant phenotypes are generated, whereas the evolution and ecology of the fig system can inform how population-level and environmental forces sort said variation. This all begins with a simple understanding of where and how this organism lives in nature.

## Additional files

**Additional file 1.** 2016 *Ficus septica* fig field data.

**Additional file 2.** Top BLAST hits for *Ficus septica* fig-derived COI sequences.

**Additional file 3.** Alignment of *Ficus septica* fig-derived COI sequences plus additional COI sequences from relevant species.

**Additional file 4: Supplemental Figure and Tables. Figure S1.** The distribution of *Ceratosolen* pollinating foundress wasps among pollinated and unpollinated *Ficus septica* figs. **Table S1.** Differences in *C. inopinata* plant occupancy among different islands Fisher’s exact test p-values. **Table S2.** Differences in *C. inopinata* fig occupancy (including pollinated figs) among different islands Fisher’s exact test p-values. **Table S3.** Differences in *C. inopinata* fig occupancy (excluding pollinated figs) among different islands Fisher’s exact test p-values. **Table S4.** *C. inopinata* occupancy in *Ficus septica* figs in 2015. **Table S5.** Differences in *C. inopinata* plant and fig occupancy in different field seasons Fisher’s exact test p-values. **Table S6.** Differences in *C. inopinata* fig occupancy given different foundress number Fisher’s exact test p-values. **Table S7.** Differences in the presence of reproductive stage *C. inopinata* given different fig stages Fisher’s exact test p-values. **Table S8.** Differences in the presence of dauer stage *C. inopinata* given different fig stages Fisher’s exact test p-values. **Table S9.** Repeated convergence of fig-association in nematodes.
