# Supplementary material for: Field studies reveal a close relative of C. elegans thrives in the fresh figs of Ficus septica and disperses on its Ceratosolen pollinating wasps
Source: BMC Ecol. 2018 Aug 21;18:26. doi: 10.1186/s12898-018-0182-z (PMC6102938; doi:10.1186/s12898-018-0182-z)
Supplement: Supplementary file 4 — Additional file 4: Supplemental Figure and Tables. Figure S1. The distribution of Ceratosolen pollinating foundress wasps among pollinated and unpollinated Ficus septica figs. Table S1. Differences in C. inopinata plant occupancy among different islands Fisher’s exact test p-values. Table S2. Differences in C. inopinata fig occupancy (including pollinated figs) among different islands Fisher’s exact test p-values. Table S3. Differences in C. inopinata fig occupancy (excluding pollinated figs) among different islands Fisher’s exact test p-values. Table S4. C. inopinata occupancy in Ficus septica figs in 2015. Table S5. Differences in C. inopinata plant and fig occupancy in different field seasons Fisher’s exact test p-values. Table S6. Differences in C. inopinata fig occupancy given different foundress number Fisher’s exact test p-values. Table S7. Differences in the presence of reproductive stage C. inopinata given different fig stages Fisher’s exact test p-values. Table S8. Differences in the presence of dauer stage C. inopinata given different fig stages Fisher’s exact test p-values. Table S9. Repeated convergence of fig-association in nematodes. [file 12898_2018_182_MOESM4_ESM.pdf]

Supplemental Figures and Tables

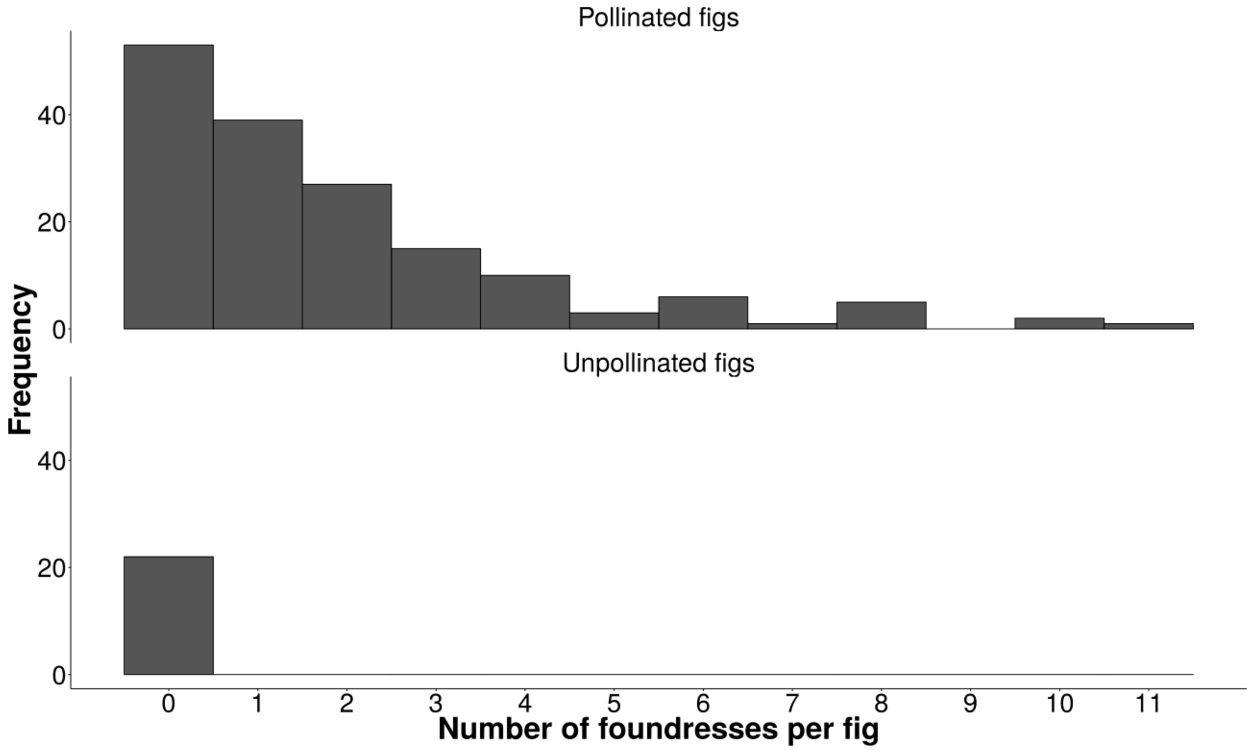

Figure S1. The distribution of *Ceratosolen* pollinating foundress wasps among pollinated (top panel) and unpollinated (bottom) *Ficus septica* figs. All unpollinated figs were assumed to not have foundresses in this panel; of 22 unpollinated figs, foundress number was only recorded for seven figs (zero foundresses in all cases). Figs without developing seeds were assumed to be unpollinated.

Table S1. Differences in *C*. sp. 34 plant occupancy among different islands Fisher's exact test p-values.

	Ishigaki	Iriomote	Yonaguni
**Miyako**	0.72	0.44	1
**Ishigaki**		0.093	1
**Iriomote**			0.088

Table S2. Differences in *C*. sp. 34 fig occupancy (including pollinated figs) among different islands Fisher's exact test p-values.

	Ishigaki	Iriomote	Yonaguni
**Miyako**	1	<0.0001	0.0051
**Ishigaki**		0.00035	0.039
**Iriomote**			0.21

Table S3. Differences in *C*. sp. 34 fig occupancy (excluding pollinated figs) among different islands Fisher's exact test p-values.

	Ishigaki	Iriomote	Yonaguni
**Miyako**	0.6323	0.00057	0.071
**Ishigaki**		0.00035	0.039
**Iriomote**			0.21

Table S4. *C.* sp. 34 occupancy in *Ficus septica* figs in 2015.

	**Iriomote**	**Ishigaki**	**Total**
**Number of plants sampled**	11	14	25
**Number of plants with *C.* sp. 34**	9	11	20
**Fraction of plants with *C.* sp. 34**	0.82	0.79	0.80
**Number of figs sampled**	62	74	136
**Number of figs with *C.* sp. 34**	28	26	54
**Fraction of figs with *C.* sp. 34**	0.45	0.35	0.40

Figs were sampled from the Okinawan islands of Iriomote and Ishigaki in May 2015. The presence of *C.* sp. 34 animals in fresh figs was ascertained via fig dissection and subsequent microscopy. This data includes all plants and figs sampled, regardless of worm occupancy and pollination status.

Table S5. Differences in *C.* sp. 34 plant and fig occupancy in different field seasons (2015 and 2016) Fisher's exact test p-values.

	**Iriomote**	**Ishigaki**
**Plant**	0.69	0.0022
**Fig**	0.62	0.12

Table S6. Differences in *C.* sp. 34 fig occupancy given different foundress number Fisher's exact test p-values.

	**0 (pollinated)**	**1**	**2**	**3-11**
**0 (unpollinated)**	0.016	0.002221	<0.0001	<0.0001
**0 (pollinated)**		0.24	0.0066	<0.0001
**1**			0.20	0.0090
**2**				0.46

Table S7. Differences in the presence of reproductive stage *C.* sp. 34 given different fig stages Fisher's exact test p-values.

	Fig Stage 2	Fig Stage 3	Fig Stage 4	Fig Stage 5
**Fig Stage 1**	<0.0001	0.0491	1	1
**Fig Stage 2**		0.1099	0.0039	<0.0001
**Fig Stage 3**			0.14	0.022
**Fig Stage 4**				1

Table S8. Differences in the presence of dauer stage *C.* sp. 34 given different fig stages Fisher's exact test p-values.

	Fig Stage 2	Fig Stage 3	Fig Stage 4	Fig Stage 5
**Fig Stage 1**	1	0.0015	<0.0001	0.022
**Fig Stage 2**		<0.0001	<0.0001	<0.0001
**Fig Stage 3**			0.16	0.36
**Fig Stage 4**				0.029

Table S9. Repeated convergence of fig-association in nematodes.

Genus/Clade	Higher-rank taxon	Phylogenetic evidence	Fig-association evidence
Schistonchus	Aphelenchoidid	[33, 38]	Multiple, [33, 38]
Parasitodiplogaster-Teratodiplogaster	Diplogastrid	[88]	Multiple, [34-37]
Ficotylus	Tylenchid	[39-40]	[39-40]
Ficophagus	Aphelenchoidid	[33, 38]	Multiple, [33, 38]
Martininema	Aphelenchoidid	[33, 38]	Multiple, [33, 38]
Bursaphelenchus	Aphelenchoidid	[38]	[38]
Pristionchus	Diplogastrid	[88]	[41]
Acrostichus	Diplogastrid	[88]	[41]
Caenorhabditis	Eurhabditis	[47-48]	This paper, [47-48]

A literature review reveals the independent evolution of fig-association in nematodes. Each row represents a lineage that evolved to thrive in figs independently.
